# *Lactiplantibacillus plantarum* Y42 in Biofilm and Planktonic States Improves Intestinal Barrier Integrity and Modulates Gut Microbiota of Balb/c Mice

**DOI:** 10.3390/foods11101451

**Published:** 2022-05-17

**Authors:** Lijuan Zhang, Yuan Meng, Jiayi Li, Jiang Yu, Guangqing Mu, Yanfeng Tuo

**Affiliations:** 1 School of Food Science and Technology, Dalian Polytechnic University, Dalian 116034, China; 15386843201@163.com (L.Z.); my_2311675814@163.com (Y.M.); 18406593038@163.com (J.L.); yujiang0324@163.com (J.Y.); tuoyf@dlpu.edu.cn (Y.T.); 2 Dalian Probiotics Function Research Key Laboratory, Dalian Polytechnic University, Dalian 116034, China

**Keywords:** biofilm, planktonic, *Lactobacillus plantarum*, intestinal barrier, gut flora

## Abstract

In our previous study, *Lactiplantibacillus plantarum* Y42 showed some potential probiotic functions and the ability to form biofilm. The aim of this study was to compare the similarities and differences in the probiotic and physiological traits of *L. plantarum* Y42 in the biofilm and planktonic states. *L. plantarum* Y42 in the biofilm state was proven to have higher survival after passing through mimic gastrointestinal fluid, as well as excellent adhesion properties on the HT-29 cell monolayers, than those in the planktonic state. The expression of tight junction proteins (TJ proteins) of HT-29 cell monolayers treated by *L. plantarum* Y42 in the planktonic state increased, while similar changes were not observed in the HT-29 cells treated by the strain in the biofilm state. Furthermore, Balb/c mice were orally administered *L. plantarum* Y42 in the biofilm and planktonic states, respectively. Compared to the planktonic state, the oral administration of *L. plantarum* Y42 in the biofilm state significantly boosted IgA levels and improved the immunity of the mice. High-throughput sequencing showed that the diversity and structure of the intestinal flora of the mice were changed after the oral administration of *L. plantarum* Y42, including the up-regulated relative abundance of *Lactobacillus* in the intestinal tract of the mice, with no difference between the biofilm and planktonic states. Moreover, oral administration of *L. plantarum* Y42 in biofilm and planktonic states reduced the release of proinflammatory factors, to a certain extent, in the serum of the mice. The similarities and differences in the probiotic and physiological properties of *L. plantarum* Y42 in the biofilm and planktonic states can be contributed to the reasonable application of the strain.

## 1. Introduction

In recent years, probiotics have been widely used as a human food additive and are widely researched for its probiotic properties [1]. Several probiotics have been demonstrated to protect the gastrointestinal tract from pathogens [2], regulate the immune system [3], alleviate diarrhea [4], and reduce cholesterol [5]; additionally, they have shown much other activity targeted to human health. It is essential that probiotic strains pass through the gastrointestinal tract and maintain high survival rate, which is considered to be one of the necessary factors for probiotics to promote their physiological functions [6,7].

Some strains belonging to lactobacilli are considered as potential probiotics and reside in human and animal intestines, mainly in the biofilm mode [8,9]. Biofilm is an aggregate of cells that are usually embedded in a self-produced extracellular polymer matrix and adhere to the surface of an organism or non-organism [10]. Lactobacilli strains have the ability to form biofilm, and bacteria cells in biofilm state have higher resistance to poor growth conditions [11]. For example, *Lactobacillus plantarum* subsp. *plantarum* JCM 1149 in biofilm lifestyle had higher survival rates to all treatments (namely organic acid, ethanol, and sodium hypochlorite) than the planktonic bacterial cells [12].

The transcriptomics and proteomics have been applied, in order to study the differences of bacteria between the biofilm and planktonic states [13,14]. It has been pointed out that the proteins and genes associated with the adhesion of the biofilm-lifestyle *Lactobacillus plantarum* stains are up-regulated, compared with planktonic bacteria [14,15]. In addition, untargeted metabolomic measurements showed significant differences in the metabolism of *B. bifidum* in biofilm and planktonic states, including 64 differential metabolites and five metabolism pathways, which were aminoacyl-tRNA biosynthesis, alanine, aspartate and glutamate metabolism, nitrogen metabolism, citrate cycle (TCA), and arginine and proline metabolism [16].

Due to the differences in the metabolites of bacterial cells between the biofilm and planktonic states, there should be some differences in the functions of the strains between biofilm and planktonic states. Therefore, the purpose of this study was to compare the differences in tolerance to gastrointestinal fluid and adhesion ability on HT-29 cell monolayers between the biofilm and planktonic states of *L. plantarum* Y42. Moreover, we assessed the effects of *L. plantarum* Y42 oral administration, in different states, on colon tight junction (TJ) proteins, immune response, and intestinal flora of Balb/c mice.

## 2. Materials and Methods

### 2.1. Bacterial Stains and Culture Conditions

The *L. plantarum* Y42 in this study was stored in the Dalian Probiotics Function Research Key Laboratory. *L. plantarum* Y42 was cultured at 37 °C for 24 h in de Man, Rogosa, and Sharpe (MRS) broth (Land Bridge Technology, Beijing, China).

### 2.2. Biofilm Formation of L. plantarum Y42

The biofilm formation of *L. plantarum* Y42 was measured using the methods, with some modifications, described by Stepanovic [17]. The concentration of *L. plantarum* Y42 was adjusted to 2 × 10^6^ CFU/mL with fresh MRS medium. A total of 100 µL of the bacteria suspension was then placed into an optically clear 96-well plastic plate (Costar 3599, Corning, NY, USA), with a negative control containing 100 μL MRS medium. The total biofilm was quantified using the crystal violet (CV) assay, after the microtiter plates were incubated for 24 h at 37 °C. First, the medium was removed, and each well was washed four times with 200 μL sterile phosphate buffer saline (PBS) to remove unattached cells. After that, 200 μL per well of 95% methanol was added to each well of the plate, in order to fix the cells for 15 min. The biofilm was stained with 200 μL of 2.0% crystal violet solution for 5 min after the methanol had evaporated and then washed with distilled water. Finally, 33% acetic acid was added for 30 min to dissolve the biofilm with the dye attached, and the optical density (OD) at 570 nm was determined by Multiskan GO 1510 (Thermo Fisher Scientific, MA, USA).

According to Sun et al.’s report, biofilm formation capacity of strains were classified as follows: OD ≤ ODc non-adherent; ODc < OD ≤ 2 × ODc weakly adherent; 2 × ODc < OD ≤ 4 × ODc moderately adherent; 4 × ODc < OD strongly adherent [15]. The ODc value was the sum of the average value and three times of the standard deviation of the blank optical density.

### 2.3. Biofilm Growth Curve and Observation by SEM

*L. plantarum* Y42 with 2% inoculation (*v*/*v*) was inoculated into fresh MRS. The microtiter plates filled with 100 µL of *L. plantarum* Y42 bacteria suspension were incubated for 8, 12, 14, 20, and 24 h at 37 °C, and biofilm formation was quantified using CV assay, as described above.

The morphologies of *L. plantarum* Y42 cultured for 8 and 20 h on sterile glass coverslips were observed according to Deng’s method, with some modifications [18]. Coverslips were immersed in each well of the 6-well polystyrene plates containing 2 mL suspension of mixed bacterial. After incubation at 37 °C for the desired time, the coverslips were aseptically removed with sterile forceps and rinsed with sterile PBS. The specimens were first fixed using 2.5% glutaraldehyde for 6–8 h, then dehydrated in a series of graded aqueous ethanol solutions and, finally, air dried at room temperature. After gold spraying, the samples were observed by SEM.

### 2.4. Preparation of Planktonic and Biofilm Cells

Planktonic and biofilm cells of *L. plantarum* Y42 were prepared according to the method reported by Sun et al. and Sadiq et al. [15,16]. According to the formation curve of biofilm of the strain, we selected the strains cultured for 8 h at 37 °C under static conditions as the planktonic state; correspondingly, biofilm cells were formed at 37 °C for 20 h under static conditions. After incubation, cells were harvested from MRS broth cultures by centrifugation (6000× *g* for 5 min at 4 °C). Finally, cell pellets were washed twice with phosphate-buffered saline (PBS), and the harvested pellets were resuspended in PBS at 10^9^ CFU/mL for subsequent experiments.

### 2.5. Tolerance to Artificial Gastrointestinal Conditions

Evaluating the gastrointestinal tolerance capacity of *L. plantarum* Y42 in planktonic and biofilm states was highly important, as they may have different effects in humans. The gastrointestinal tolerance of strains was previously evaluated by Zhou et al. [19]. For the preparation of artificial simulated gastric juice, 0.3% pepsin (*w*/*v*) was dissolved in phosphate buffer solution; then, the pH was adjusted to 2.5. Additionally, the phosphate buffer solution containing 1.8% bile salt (*w*/*v*) and 0.3% trypsin (*w*/*v*) was prepared; then, the pH was adjusted to 8.0, which is known as artificial simulated intestinal juice. The 50 μL pre-cultured strains (1 × 10^9^ CFU/mL), in the biofilm and planktonic states, were inoculated in 450 μL simulated gastric juice and cultured at 37 °C for 3 h; then, 50 μL culture medium was inoculated at 450 μL simulated intestinal fluid and cultured at 37 °C for 8 h. The survival number of bacteria in the simulated gastric juice and simulated intestinal fluid was determined via the plate counting method, using MRS agar medium. The survival rate of the strains was evaluated by the following equation:

$$\mathrm{Survival}\mathrm{rate}\;(\%)=\frac{\mathrm{Cell}\mathrm{number}\mathrm{after}\mathrm{ncubation}\;\left(\mathrm{Log}\;\mathrm{CFU}/\mathrm{mL}\right)}{\mathrm{Initial}\mathrm{cell}\mathrm{number}\;\left(\mathrm{Log}\;\mathrm{CFU}/\mathrm{mL}\right)\;}\times 100$$

### 2.6. HT-29 Cell Culture and Adhesion Assay on HT-29 Cells of Biofilm and Planktonic Bacteria

#### 2.6.1. HT-29 Cell Culture

The human colon cancer cell line HT-29 was obtained from the Chinese Academy of Science (Shanghai, China). The HT-29 cells were cultivated in RPMI-1640 medium supplemented with 10% (*v*/*v*) (56 °C, 30 min) fetal calf serum (Sijiqing Co., Ltd., Hangzhou, China) at 37 °C, with 5% CO_2_ and 95% air [20]. Then, cell viability was assessed using trypan-blue dye (0.2%) in PBS (pH 7.2), and the cell number was determined by hemocytometer.

#### 2.6.2. Adhesion Assay on HT-29 Cells of Biofilm and Planktonic Bacteria

The adhesion assay was performed as described by Greppi, with minor modifications [21]. Briefly, HT-29 cells were seeded at 2.5 × 10^5^ cells per well in 24-well microtiter plates and cultured above for 2–4 days. Then, the cells were washed twice with PBS, in order to remove antibiotics. *L. plantarum* Y42 in planktonic and biofilm states were resuspended in RPMI-1640 medium, without antibiotics, up to 1 × 10^8^ CFU/mL, respectively. A total of 100 μL of the *L. plantarum* Y42 suspensions were transferred into the wells and incubated for 2 h at 37 °C. To remove unattached bacteria, the HT-29 cells were washed three times with PBS. Afterwards, 500 µL of Triton-X solution (5% in PBS) was added into the wells to digest HT-29 cells. Then, the mixtures in each well were homogenized. The mixture solution was continuously diluted, and the *L. plantarum* Y42 number attached to the HT-29 cells was counted pouring plate method on MRS agar. The adhesion ability of *L. plantarum* Y42 was evaluated by the following equation:

$$\mathrm{Adhesion}\mathrm{ability}\;(\%)=\frac{\mathrm{Attached}\mathrm{number}\mathrm{of}\mathrm{bacterial}\;(\mathrm{CFU}/\mathrm{mL})}{\mathrm{Initial}\mathrm{number}\mathrm{of}\mathrm{bacterial}\;(\mathrm{CFU}/\mathrm{mL})}\times 100$$

### 2.7. Animal and Experimental Design

All animal experiments were carried out in accordance with the guidelines of the Experimental Animal Ethics Committee of Dalian Polytechnic University (SYXK2017-0005). Seven-week-old Balb/c female mice (~20 g) were used in the study. They were purchased from Liaoning Changsheng Biotechnology Company (Benxi, China) and allowed to acclimate for one week prior to experiment; they were provided with fresh water and commercial food. The mice were then randomly divided into three experimental groups, with six animals each group, which were expressed as the con, planktonic (oral administered with planktonic cells of *L. plantarum* Y42), and biofilm groups (oral administered with biofilm cells of *L. plantarum* Y42). Afterwards, the cells were washed twice in sterile physiological saline and suspended in sterile physiological saline solution to a final concentration of 10^9^ CFU/mL. The planktonic and biofilm groups were orally administered to the mice once daily, respectively, at a dose of 0.2 mL (10^9^ CFU), for a period of 14 d.

The mice were deprived of food and water for 12 h, then anesthetized, and blood was collected from the retrobulbar plexus of mice on the 14th d. After centrifugation (2000× *g*, 10 min, 4 °C), a serum sample was collected. The colons were separated from the proximal rectum, close to its passage under the pelvisternum.

### 2.8. IgA, IgG, IgM, IL-6, TNF-α, and IL-10 Analysis

Serum samples were obtained from blood of mice by centrifugation at 2010× *g* for 10 min at 4 °C and stored at −80 °C after freezing in liquid nitrogen. The concentrations of IgA, IgG, IL-6, TNF-α, and IL-10 in the serum of the mice were detected by ELISA kits (Nanjing Jiancheng Bioengineering Institute, Nanjing, China), according to the instructions of the manufacturer.

### 2.9. Western Blotting Analysis

The western blotting analysis was performed as described by Fu et al., with minor modifications [4]. Total proteins were obtained from mice colon tissue of the mice and HT-29 cells by protein extraction kit (Solarbio Life Science, Beijing, China), according to manufacturer’s description. The concentration of protein was quantified using the BCA protein assay kit (Solarbio Life Science, Beijing, China). Next, the equivalent protein samples were subjected to electrophoresis using sodium dodecyl sulfate (SDS)-polyacrylamide gel electrophoresis (PAGE), and electrophoresis was stopped just after the electrophoretic band of bromophenol blue ran out. After electrophoresis was completed, the proteins were transferred to polyvinylidene difluoride (PVDF) membranes, and the PVDF membranes were sealed with 5% skim milk powder for 1 h. The skim milk powder on the PVDF was washed using TBST (five times each time) for 10 min. Then, the PVDF membranes were incubated overnight at 4 °C with primary antibodies, including occludin, claudin-1, and ZO-1 (1:2000, Beyotime Institute of Biotechnology, Shanghai, China). After washing with TBST (5 times each time) for 10 min, the membranes were warmed with secondary antibody (1:2000, Beyotime Institute of Biotechnology, Shanghai, China). Finally, the specific bands were visualized with the ECL detection kit (Beyotime Institute of Biotechnology, Shanghai, China), and the bands were visualized for quantification using ImageJ software (National Institutes of Health, Bethesda, MD, USA).

### 2.10. Gut Microbiota Analysis

Fresh feces of the mice were frozen with liquid nitrogen immediately after collection and kept at −80 °C. The genomic DNA of the sample was extracted by the cetyltrimethylammonium bromide method. Briefly, the samples were added to CTAB buffer, and the total DNA was extracted with chloroform: isoamyl alcohol (24:1), and the precipitation was washed twice with 75% ethanol and dissolved with ddH_2_O. Then, the purity and concentration of DNA were detected by 1% gel electrophoresis. All PCR reactions were carried out with 15 µL of Phusion^®^ High-Fidelity PCR Master Mix (New England Biolabs, Ipswich, MA, USA), 2 µM of forward and reverse primers, and about 10 ng template DNA. Mixture PCR products was purified with Qiagen gel extraction kit (Qiagen, Dusseldorf, Germany). Sequencing libraries were generated using TruSeq^®^ DNA PCR-Free Sample Preparation kit (Illumina, San Diego, CA, USA), following manufacturer’s recommendations, and index codes were added. HTS was performed using an Illumina HiSeq platform (Novogene Bioinformation Science and Technology Co., Ltd., Beijing, China).

### 2.11. Statistical Analysis

Results were expressed as the mean ± SD. Statistical analysis were performed using GraphPad Prism software (version 8.2.1) and repeated-measures ANOVA, with the level of significance set at *p* < 0.05.

## 3. Results and Discussion

### 3.1. Biofilm Formation of L. plantarum Y42

The biofilm formation of *L. plantarum* Y42 was determined by the CV assay. *L. plantarum* Y42 adhered on polystyrene and formed biofilm after 24 h growth, having a moderate capacity for biofilm formation (OD_570_ mean value = 0.56). Sun et al. estimated the biofilm formation ability of 79 strains of lactic acid bacteria (LAB) using CV assay, with the OD_570nm_ values ranging from 0.412 ± 0.054 to 2.266 ± 0.057, and found that different strains had different biofilm-forming abilities, which may be due to the species specificity of the strains [15].

**Figure 1:**
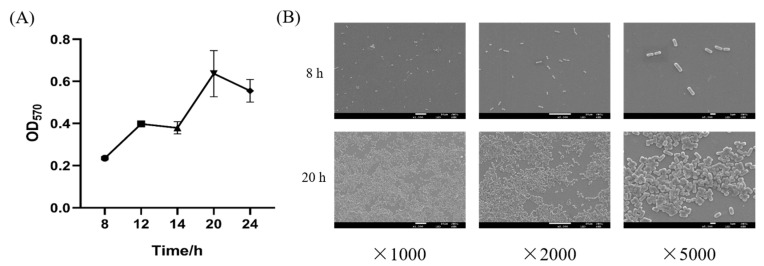
Biofilm formation curve of *L. plantarum* Y42 (**A**); SEM images of the *L. plantarum* Y42 biofilms on glass at 8 and 20 h (**B**).

As shown in Figure 1A, biofilm formation could be affected by culture time. When culture time reached 8 h, the initial adhesion of *L. plantarum* Y42 on polystyrene started, but *L. plantarum* Y42 biofilm was not obvious. As the culture time extended to 20 h, the mature biofilm of *L. plantarum* Y42 on polystyrene was formed. During the period from 20 to 24 h, the total biofilm decreased, due to the mature biofilm falling off.

In addition, SEM was employed to observe the morphologies of the *L. plantarum* Y42 on glass coverslips at 8 and 20 h. As shown in Figure 1B, *L. plantarum* Y42 existed only as a single or paired bacterium at 8 h and was considered as a planktonic state. At 20 h, *L. plantarum* Y42 cells aggregated together and stuck to the coverslips, indicating the matured biofilm at 20 h.

Therefore, *L. plantarum* Y42 cells cultured for 8 h were selected as a planktonic state, and *L. plantarum* Y42 cells cultured for 20 h were selected as a biofilm state.

### 3.2. Tolerance of Biofilm and Planktonic L. plantarum Y42 to Artificial Gastrointestinal Conditions

To test the tolerance of the *L. plantarum* Y42 in the different states under the human gastrointestinal tract, the viability of the *L. plantarum* Y42 under the artificial gastrointestinal conditions was evaluated. As shown in Table 1, compared with initial cell numbers, under the acid stress of gastric fluid, the survival rates of *L. plantarum* Y42 in different states showed no significant difference. Then, *L. plantarum* Y42 in the biofilm state showed a significantly higher (*p* < 0.05) survival rate when exposed to simulated intestinal fluid than that in the planktonic state, indicating that *L. plantarum* Y42 in the biofilm state had greater resistance to gastrointestinal conditions.

The survival of bacteria in the gastrointestinal tract is important for the physiological functions of probiotics [22]. *L. plantarum* Y42 in the biofilm state showed a relatively stronger resistance to artificial gastrointestinal conditions. *L. plantarum* (No. 23941) in the biofilms showed excellent gastrointestinal resistance, as compared with planktonic cells [23], which was consistent with our present results. It was reported that the heat shock proteins and some amino acid biosynthetic pathways of *L. plantarum* J26 in the biofilm state were increased, which enhanced the ability of *L. plantarum* J26 to resist environmental stress [15]. In addition, extracellular matrix secreted by *L. paraplantarum* L-ZS9, such as polysaccharides and proteins, are important to withstand external pressure [24].

### 3.3. Adhesion Rates of Biofilm and Planktonic L. plantarum Y42 on HT-29 Cell Monolayers

**Figure 2:**
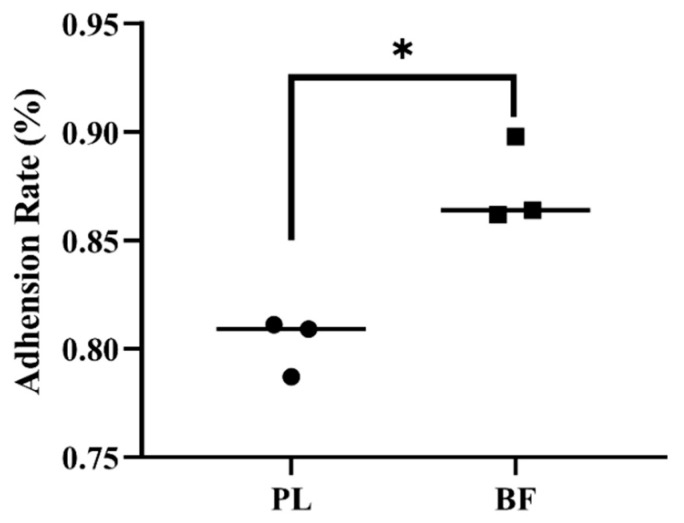
Adhesion rates of *L. plantarum* Y42 in the biofilm (BF) and planktonic (PL) states on HT-29 cell monolayers; *: *p* < 0.05. All data are presented as mean ± SD.

Adhesion on the intestinal epithelium of the host is an important profile for probiotics [2]. As shown in Figure 2, the adhesion rate of *L. plantarum* Y42 in the biofilm state on HT-29 cell monolayers was 1.1 times higher than that of the planktonic state. It was proven that *L. plantarum* DB200 in the biofilm state excreted more adhesion-related proteins than that of the planktonic state and showed the highest autoaggregation [14].

### 3.4. Effects of Biofilm and Planktonic L. plantarum Y42 on the TJ Proteins Expression of HT-29 Cell Monolayers

**Figure 3:**
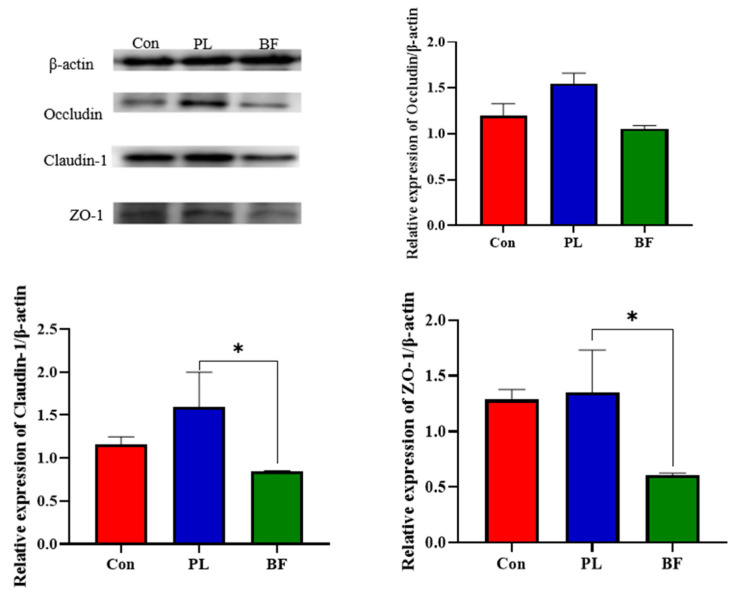
Effect of *L. plantarum* Y42 in the planktonic and biofilm states administration on the TJ proteins expression in the HT-29; *: *p* < 0.05. All data are presented as mean ± SD. Note: Con means control group; PL means planktonic group; BF means biofilm group.

To evaluate the effects of *L. plantarum* Y42 in the biofilm and planktonic states on barrier function of the HT-29 cell monolayers, the expression of TJ proteins (claudin-1, occludin, and ZO-1) was assessed by western blotting. As shown in Figure 3, compared to the BF group, the relative expression of claudin-1 and ZO-1 of the HT-29 cell monolayers in the PL group was significantly up-regulated. Similarly, the expression of occludin was also up-regulated to certain levels in the PL groups, but was not significantly different to the BF group.

TJ proteins play an important role in maintaining the intestinal barrier function, which are in dynamic change under the stimulation of external substances [25]. TJ proteins separate coelenterates from their internal environment and limit the transmission of pathogens, toxins, and allergens as a physical barrier. In the present study, *L. plantarum* Y42 in the planktonic state increased the expression of TJ proteins, and we concluded that planktonic *L. plantarum* Y42 enhanced the intestinal barrier function, compared to biofilm *L. plantarum* Y42. It was reported that there were significant differences in amino acid metabolic pathways of *L. paraplantarum* L-ZS9 in the biofilm and planktonic states, and the contents of arginine, proline, and L-valine in the biofilm cells were increased, while only L-glutamine was increased in planktonic cells [23]. Glutamine is an important nutrient in the metabolism of small intestines and maintains the intestinal mucosal barrier by increasing the height of the intestinal villi, reducing the permeability of the intestinal mucosa, and preventing bacterial migration [26,27]. Several studies have shown that the extracellular matrix of bacteria biofilm, such as extracellular polysaccharide, which limits the communication between the environment and bacteria cells interior and prevents the bacteria from binding to the cell receptor toll-like signaling (TLR) of the intestinal epithelial cells [28]. TLR signaling mediates the TJ proteins’ changes in colon tissues after feeding of *L. plantarum* WCFS1 [29]. Compared to the planktonic *L. plantarum* Y42, *L. plantarum* Y42 in the biofilm state down-regulated the tight junction proteins expression. The explanation for this is unknown, but it might be due to biofilm *L. plantarum* Y42 impacting ZO-1 and occludin translocation in cells [30].

### 3.5. Animal Experiments

#### 3.5.1. Effects of Biofilm and Planktonic *L. plantarum* Y42 Administration on Body Weight of Balb/c Mice

**Figure 4:**
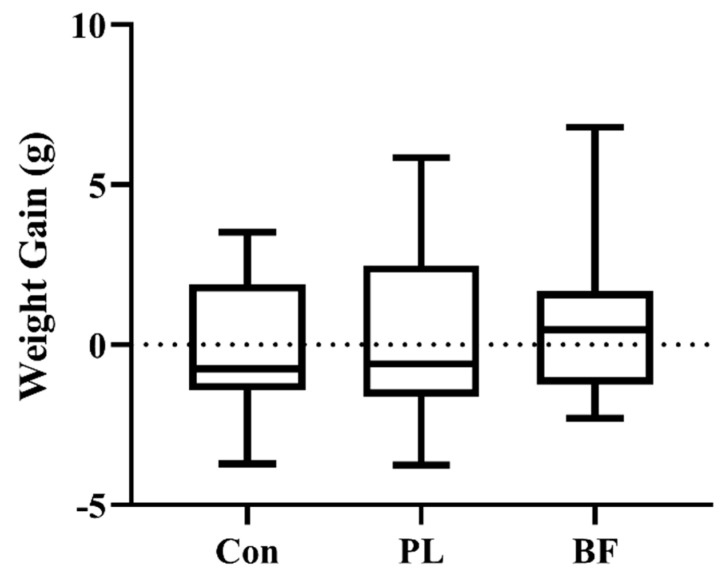
Effects of *L. plantarum* Y42 in the planktonic (PL) and biofilm (BF) states on weight gain in mice. All data are presented as mean ± SD (*n* = 6 mice per group). Note: Con means control group; PL means planktonic group; BF means biofilm group.

Furthermore, the *L. plantarum* Y42 in the biofilm and planktonic states was orally administered to Balb/c female mice, respectively. The mice did not experience any negative effects as a result of the strain oral treatment, and the change of body weight was also measured daily during *L. plantarum* Y42 oral administration. As shown in Figure 4, the weight gains of the Balb/c mice among the three groups showed no significant difference (*p* > 0.05). Each experimental group of mice gained normal weight, indicating that *L. plantarum* Y42 is safe for animal growth.

#### 3.5.2. Effects of Biofilm and Planktonic *L. plantarum* Y42 Administration on Immunity of Balb/c Mice

**Figure 5:**
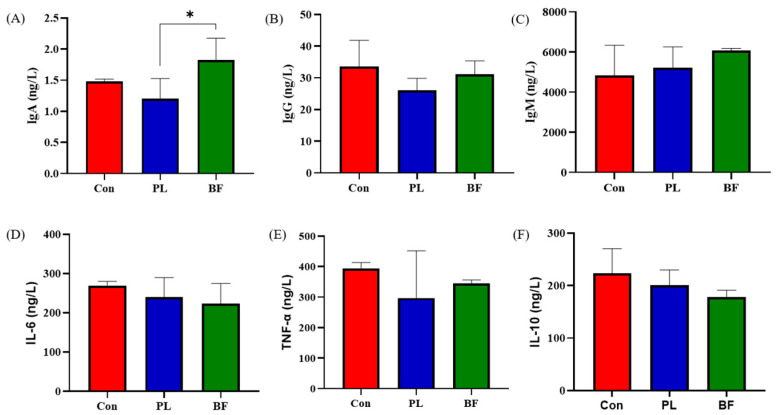
The concentration of (**A**) IgA, (**B**) IgG, (**C**) IgM, (**D**) IL-6, (**E**) TNF-α, and (**F**) IL-10 in the blood samples from mice at the end of the animal experiment (14th d); *: *p* < 0.05. All data are presented as mean ± SD. Note: Con means control group; PL means planktonic group; BF means biofilm group.

Probiotics could increase the serum immunoglobulin contents of the host [31]. In order to evaluate the effects of *L. plantarum* Y42 in the biofilm and planktonic states on the immunology in the serum of mice, the contents of IgA, IgG, and IgM were measured. As shown in Figure 5A–C, the concentration of IgA in BF group was significantly higher than PL group (*p* < 0.05). Additionally, the IgG and IgM concentrations in the three groups showed no significant differences (*p* > 0.05). These results indicated that the concentrations of immunoglobulin in both groups remained relatively normal, and the addition of probiotics moderated and prevented an excessive increase in immunoglobulin.

During the experiment, the levels of IL-6, IL-10, and TNF-α in the three groups were similar, indicating that the *L. plantarum* Y42 in biofilm and planktonic states did not cause an inflammatory response in the healthy Balb/c mice. A previous study showed that *P. pentosaceus* LI05 significantly reduced the serum proinflammatory cytokine levels, which is not in line with our results [32]. However, research showed that excessive amounts of proinflammatory cytokines could cause intestinal tissue injury and damage the body’s immune balance [33]. An appropriate amount of proinflammatory cytokines could regulate the immune response, resist or eliminate pathogen infection, promote the repair of damaged tissues, and cause tumor cell apoptosis [34].

#### 3.5.3. Effects of Biofilm and Planktonic *L. plantarum* Y42 Administration on the TJ Proteins Expression of Balb/c Mice

**Figure 6:**
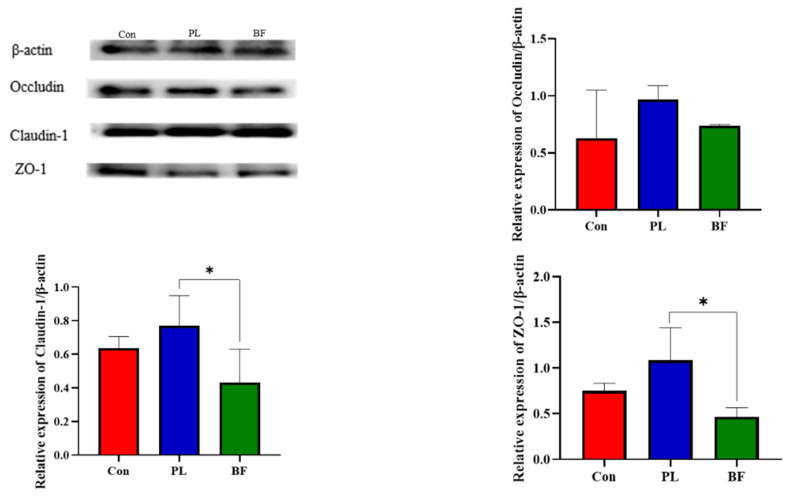
Effect of *L. plantarum* Y42 in the planktonic and biofilm states administration on the TJ proteins expression in mice; *: *p* < 0.05. All data are presented as mean ± SD. Note: Con means control group; PL means planktonic group; BF means biofilm group.

Furthermore, to further verify that effects of *L. plantarum* Y42 in the biofilm and planktonic states on intestinal barrier function, TJ proteins expression in the Balb/c mice were measured. We found that *L. plantarum* Y42 in the planktonic state significantly promoted the expression of the TJ proteins ZO-1, claudin-1, and occludin in the colon of the mice (Figure 6), which was consistent with the results in vitro. The effects of probiotic in different states on intestinal barrier function has rarely been studied. Thus, the specific causes of this phenomenon are not clear and will require additional research. As discussed above, the differences in amino acid metabolic pathways and biofilm matrix of probiotic strains in the biofilm and planktonic states may account for the phenomenon.

#### 3.5.4. Effects of Biofilm and Planktonic *L. plantarum* Y42 Administration on the Gut Microbiota of Balb/c Mice

**Figure 7:**
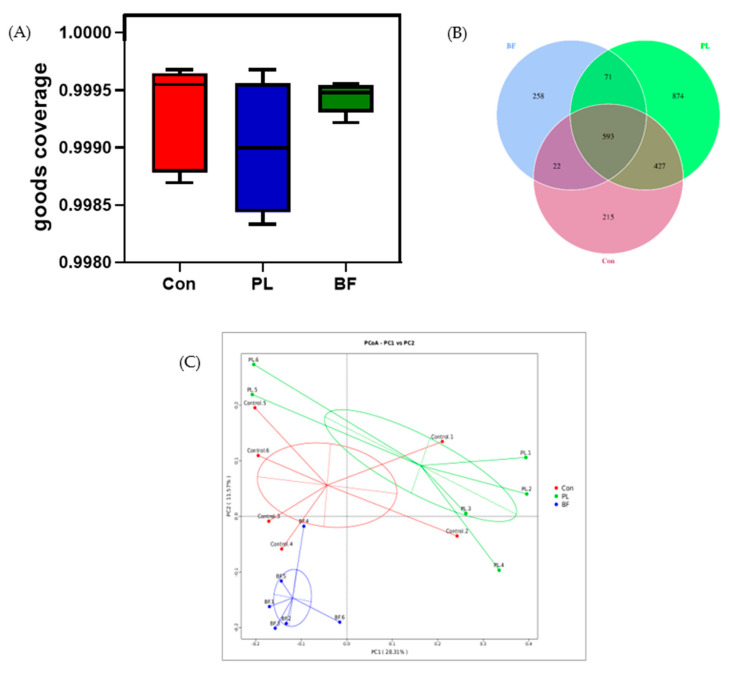
Goods coverage (**A**); OTUs Venn digram (**B**); PCoA plot, based on unweighted unifrac cluster (PCoA) of the data set (**C**). Note: Con means control group; PL means planktonic group; BF means biofilm group.

The microbial composition of feces in the Balb/c mice was analyzed by 16S rDNA sequencing. Firstly, the overall structural changes of intestinal flora were profiled. As shown in Figure 7A, the goods coverage score of each group was more than 99%, indicating that the depth of the sequencing provided was adequate to the subsequent bioinformatics analysis. As shown in Figure 7B, the OTUs of the PL and BF groups, shared with the Con group, were 1020 and 615, respectively, in which 593 OTUs were shared by all groups. To evaluate the dissimilarity and community composition of three groups, a PCoA map was established. As shown in Figure 7C, compared with the Con group, the mice with oral administration of *L. plantarum* Y42 in different states formed an obvious clustering phenomenon, indicating that *L. plantarum* Y42 in the biofilm and planktonic states altered the communities of intestinal flora in a characteristic direction.

**Figure 8:**
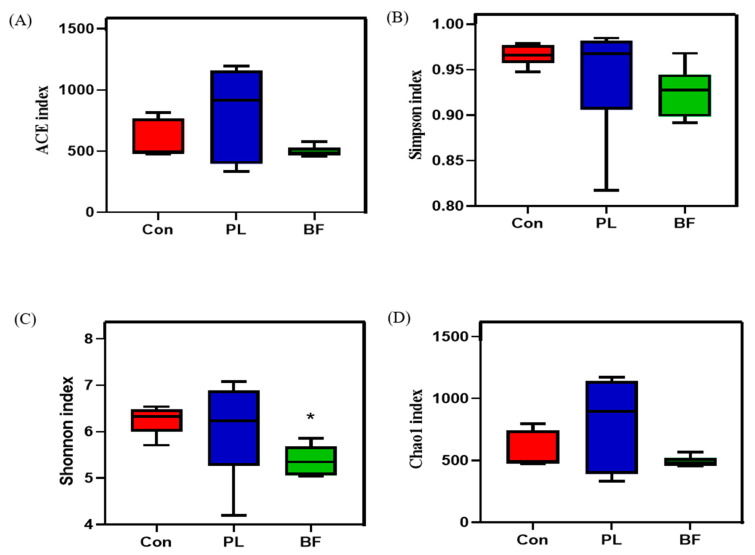
Comparison of bacterial diversity among different bacteria. ACE (**A**), Simpson (**B**), Shannon (**C**), and Chao1 (**D**) indices; *: *p* < 0.05. All data are presented as mean ± SD. Note: Con means control group; PL means planktonic group; BF means biofilm group.

The richness and diversity of intestinal microbiota were assessed by α-diversity analysis, including the Shannon, Simpson’s, Chao1, and ACE indices. As shown in Figure 8, the α -diversity (the Simpson’s, Chao1, and ACE indices) of the intestinal flora of Balb/c mice in *L. plantarum* Y42 intervention groups showed no significant difference among the PL and BF groups. However, the result of Shannon index was significantly decreased in the BF groups, compared to the PL group, suggesting that *L. plantarum* Y42 in biofilm and planktonic states could change the gut flora structure of the Balb/c mice.

**Figure 9:**
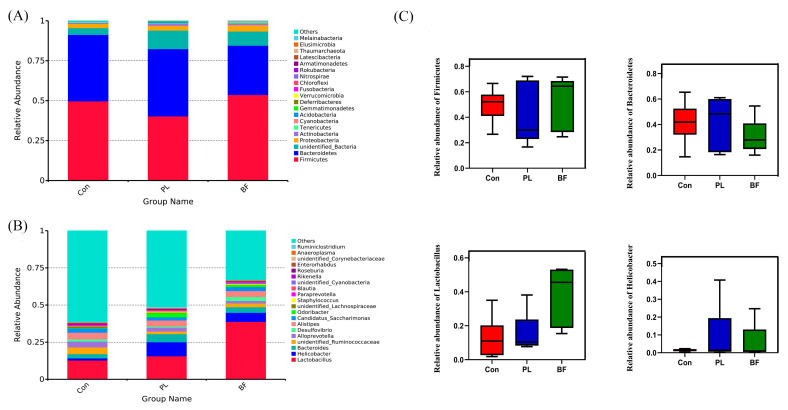
Relative abundance of bacterial communities of the fecal samples at the level of phylum (**A**), genus (**B**), and the relative abundance of Firmicutes, Bacteroidetes, Lactobacillus, and Helicobacter (**C**) of bacterial communities. All data are presented as mean ± SD. Note: Con means control group; PL means planktonic group; BF means biofilm group.

Next, the average bacterial compositional profiles were summarized at the phylum and genus levels, respectively, as shown in Figure 9. At the phylum level, the dominant phyla in three groups were *Firmicutes*, *Bacteroidetes*, *Proteobacteria*, and *Actinomycetes*. As shown in Figure 9C, the relative abundance of *Firmicutes* in the BF group was higher than in the PL group, while *Bacteroidetes* in the PL group was higher. At the genus level, compared with the Con group, *Lactobacillus* and *Helicobacter* increased in the PL and BF groups. Compared with the PL group, the relative abundance of lactobacilli in the biofilm state was higher, but not significantly different.

**Figure 10:**
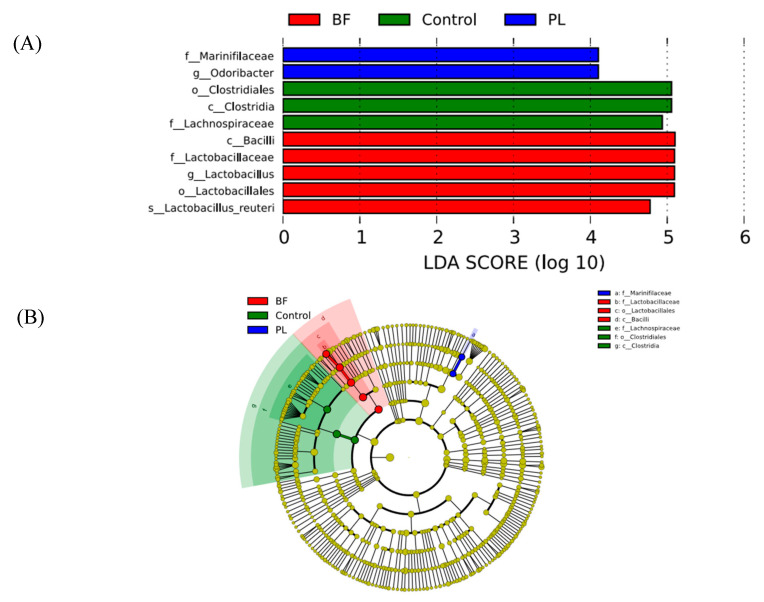
LDA scores (**A**) and Cladogram (**B**) of the fecal samples of bacterial communities. Note: PL means planktonic group; BF means biofilm group.

In order to further clarify the characteristic microorganisms of the intestinal flora in the Con and experimental groups, LEfSe analysis was carried out. According to Figure 10, there are three taxa in the Con group, five taxa in the BF group, and two taxa in the PL group. In the BF group, the characteristic microorganisms were identified as *Bacilli* (order level) and *Lactobacillales-Lactobacillaceae-Lactobacillus* (order-family-genus level). In this study, *Lactobacillus* increased in the mice intestinal tract of the BF group, indicating that the administration of *L. plantarum* Y42 in the biofilm state could increase the survival of lactobacilli in gastrointestinal tract, thus increasing its growth and adhesion. We included that the slow release of bacteria inside the biofilms and higher colonization efficiency in the intestinal tract may be the reasons why *L. plantarum* Y42 administration in the biofilm state makes lactobacilli increased in mice gut. Probiotics have a great influence on intestinal flora. Additionally, at the genus level, the relative abundance of *Helicobacter* after planktonic and biofilm *L. plantarum* Y42 administration increased. However, *Helicobacter ganmani*, which is the first anaerobic species of *Helicobacter*, decreased significantly after the intragastric administration of probiotics, which was inconsistent with our finding [35]. These results indicated that Y42 in different states could play different effects on regulating and controlling intestinal flora.

#### 3.5.5. Predicted Functional Genes in the Gut Microbiota of the Balb/c Mice

**Figure 11:**
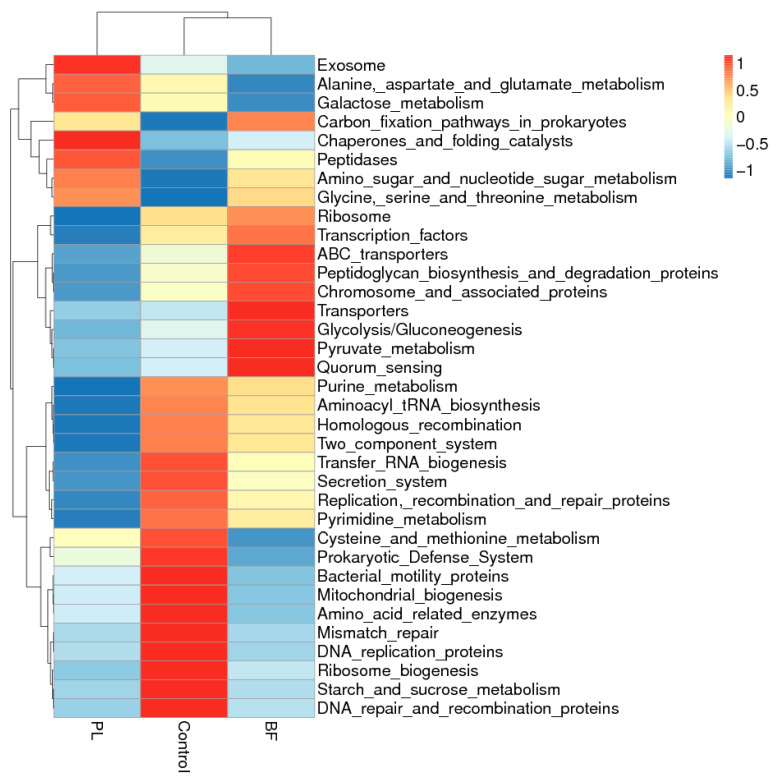
Heatmap of Tax4Fun functional prediction analysis for differential bacteria among the three groups. Note: PL means planktonic group; BF means biofilm group.

The functional profiling of the microbial communities of the mice was predicted using Tax4Fun in this study. As shown in Figure 11, to understand the underlying mechanisms of the differences in the PL and BF groups, from the function prediction of Tax4Fun, the differential functions of the bacterial genes in the three groups mainly included various metabolisms and transport. Most notably, the main metabolic functions are mainly the alanine, aspartate, and glutamate metabolisms, as well as the galactose metabolisms in the BF group. Additionally, it was shown that there were obvious differences in the ABC transporters, quorum sensing, and two-component system of the functions of the bacterial genes between the PL and BF groups. Our results revealed that quorum sensing was significantly higher in the planktonic-treated group than in the biofilm-treated group. Quorum sensing is a cell-to-cell communication process that depends on the extracellular signal molecules secreted by bacteria, called autoinducers [36]. Through a sophisticated intercellular communication network, these signal molecules drive the changes in gene expression and coordinate collective activity [37]. We hypothesized that bacteria in different states would stimulate quorum sensing among the microflora, in order to maintain the microflora’s relative stability. We need to further study the molecular mechanism regarding interactions between the microbial communities.

#### 3.5.6. Correlation Analysis between Occludin, Claudin-1, ZO-1, and Intestinal Microbiome Diversity of the Balb/c Mice

**Figure 12:**
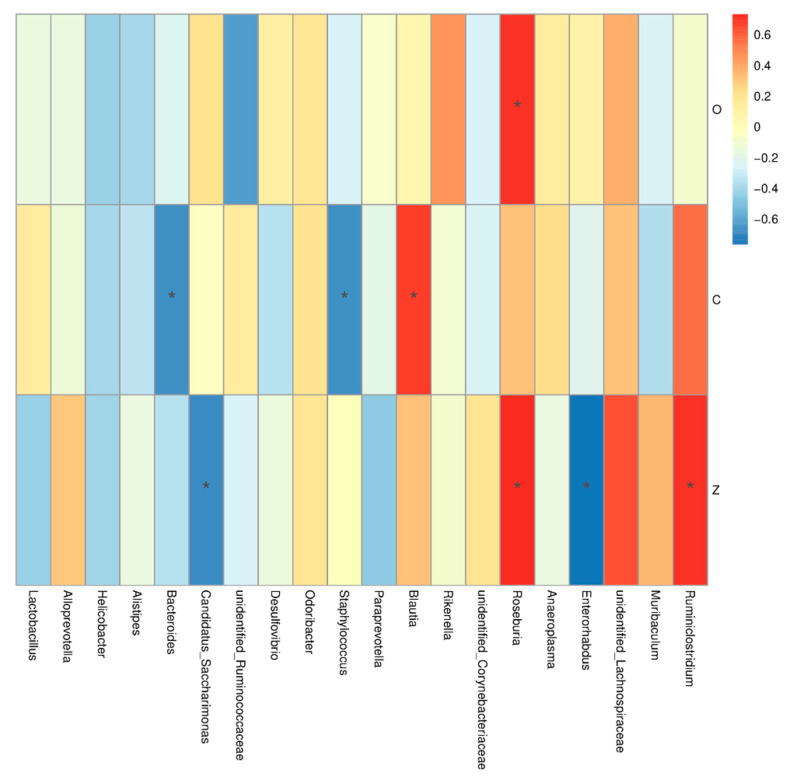
Heatmap of Spearman correlation analysis between the top 20 abundant genera of the intestinal microbiome and TJ proteins of mice. The Spearman correlation coefficient r ranges from −0.5 to 0.5; r < 0 is negative correlation; r > 0 is positive correlation; *: *p* < 0.05. Note: O means occludin; C means claudin-1; Z means ZO-1.

To further determine the relationship between the intestinal microbiome and TJ proteins, their correlation was calculated, and then the relationship was visualized as shown in Figure 12. The Spearman correlation analysis revealed the relative expression of claudin-1 was negatively correlated with the abundance of *Bacteroides* and *Staphylococcus*. Bacteroides are predominant human colonic commensals, which are closely related to the mucosal surface; however, it was found that *Bacteroides fragilis* strains can invade intestinal tissue and cause damage [38,39]. ZO-1 protein’s expression was negatively correlated with *Enterorhabdus* and *Candidatus_Saccharimonas*; however, it was positively coorelated with *Roseburia* and *Ruminiclostridium.* A previous study reported that, after severe burn injury, the relative abundance of *Roseburia* and *Ruminiclostridium* increased in mice, leading to the occurrence of intestinal barrier disruption, which was not consistent with our present results [40]. Notably, the relationships between bacterial compositions and the intestinal barrier were only mathematically predicted, and additional experimental confirmation is required.

## 4. Conclusions

The effects of *L. plantarum* Y42 in the planktonic and biofilm states on the intestinal barrier function and gut flora structure of Balb/c mice were studied. We found that *L. plantarum* Y42 in the biofilm state showed stronger resistance to mimic gastrointestinal fluid and higher adhesion rate on the HT-29 cell monolayers than the planktonic state. *L. plantarum* Y42 treatment in the planktonic state clearly increased TJ protein expression, thus maintaining the integrity of the intestinal epithelial barrier on the HT-29 cell monolayers, as well as in the colon of mice. In addition, the *L. plantarum* Y42 in the biofilm and planktonic states showed different effects on the intestinal flora in mice. High-throughput sequencing showed that *L. plantarum* Y42 in the planktonic state increased the intestinal flora diversity. *L. plantarum* Y42 increased the proportion of lactobacilli of the intestinal flora in mice, especially the biofilm state. In conclusion, although *L. plantarum* Y42 in the biofilm state could increase gastrointestinal resistance ability and adhesion rates on the HT-29 cell monolayers, *L. plantarum* Y42 in the planktonic state could promote the expression of TJ proteins and increase the diversity of the microflora. The data in this study give the similarities and differences in the probiotic and physiological properties of *L. plantarum* Y42 in the biofilm and planktonic states, which may provide a new idea for the research and development of probiotics.

## Figures and Tables

**Table 1:** Tolerance to artificial gastric and intestinal conditions of *L. plantarum* Y42 in the biofilm (BF) and planktonic (PL) states; *p* < 0.05. All data are presented as mean ± SD. Different small capital letters are presented as significantly different (*p* < 0.05).

Time/h	Initial Cell Number (Log CFU/mL)	Gastric Juice		Intestinal Juice	
		Cell Number (Log CFU/mL)	Survival Rate/%	Cell Number (Log CFU/mL)	Survival Rate/%
PL	9.19 ± 0.34	7.63 ± 0.13	83.05 ± 1.59	4.67 ± 0.38	61.20 ± 3.94 ^a^
BF	9.64 ± 0.26	7.65 ± 0.32	79.32 ± 1.22	6.70 ± 0.08	87.65 ± 2.73 ^b^

## Data Availability

The data presented in this study are available on request from the corresponding author.
